# Feeding selectivity and a functional trade-off in a benthic fish with a continuous morphological variation: an experimental test

**DOI:** 10.1186/s40850-024-00194-z

**Published:** 2024-03-04

**Authors:** Chiharu Endo

**Affiliations:** 1 Laboratory of Animal Ecology, Graduate School of Science, Kyoto University, Kyoto, Japan; 2 Present Address: Laboratory of Forest Biology, Graduate School of Agriculture, Kyoto University, Kitashirakawa Oiwake-cho, Sakyo-ku, 606-8502 Kyoto, Japan

**Keywords:** Functional trade-offs, Mouthpart morphology, Non-discrete variation, Feeding performance, Behavior, Fitness, Intermediate phenotypes

## Abstract

**Background:**

Functional trade-offs through ecological specializations are hypothesized to become causes of adaptive phenotypic divergence under divergent natural selection, where intermediate phenotypes may have the lowest fitness. Evidence of phenotypic divergence in a trade-off between populations experiencing different environmental/ecological conditions is abundant. However, traits in divergent selection sometimes present non-discrete (unimodal) variability, including intermediate phenotypes, although the underlying mechanisms are poorly documented. A benthic cyprinid fish, *Pseudogobio esocinus*, in Lake Biwa, central Japan, exhibits a large non-discrete/continuous variation in mouthpart morphology (from wide to narrow) within a lake population. The variation is linked with individual diets (i.e., the compositions of two different types of prey) even at a single site, and thus the variability is hypothesized to persist under divergent selection for prey usage. As a first step toward understanding the persistence mechanisms, here I examined the presence of morphology-dependent feeding selectivity and a functional trade-off in a laboratory experiment.

**Results:**

When each experimental fish was simultaneously provided the different types of prey (chironomid larvae and amphipods), the fish mostly utilized chironomid larvae as primary prey. However, compared with the wider-mouthed fish, the narrower-mouthed fish took a larger proportion of amphipods as secondary prey by changing feeding (attacking) behavior. The intermediate-mouthed fish had lower feeding efficiency than the extreme-mouthed fish, indicating potential disadvantage of the intermediate phenotype.

**Conclusions:**

This experimental result supports the presence of morphology-dependent feeding performance and a functional trade-off with potential impacts on trait variability, which may favor specializations rather than generalizations. In the wild, however, there may be some situations for relaxing the trade-off, such as temporally fluctuating prey availability that could also favor generalizations depending on the conditions, and thus, both extreme and intermediate phenotypes may persist/coexist in a single habitat. Although further examinations, especially focusing on feeding efficiency for each prey type separated from the effects of prey selectivity, are needed, this case represents an opportunity to consider the possible mechanisms of the persistence of phenotypic variation that is maintained without divergence even in a trade-off.

**Supplementary Information:**

The online version contains supplementary material available at 10.1186/s40850-024-00194-z.

## Background

Revealing how and why intrapopulation variability can be maintained is one of important issues in ecology and evolutionary biology [1–4]. Individuals within a population (even independent of sexes and age classes) often exhibit different ecological specializations, where they show adaptive phenotypic variation, e.g., in morphological and behavioral traits, relating differential niche using [5–8]. When a particular trait is optimized for one activity, the efficiency of that trait for another activity may be lessened as a consequence of functional constraints associated with the range of utility of the trait [9, 10]. Therefore, through ecological specializations, individuals often face functional trade-offs between traits and organismal performance [9, 11].

Functional trade-offs are hypothesized to become causes of adaptive phenotypic divergence under divergent natural selection between environments (resources), where intermediate phenotypes can be least efficient (as a jack of all trades is master of none) [9, 10, 12]. Evidence of phenotypic divergence between populations/subpopulations experiencing different environmental conditions is abundant [13]. As typical examples, in some birds, differential features in bill morphology are observed, with populations that utilize hard foods exhibiting a large, thick bill, whereas populations that utilize small/soft foods have a small bill, which is often referred to as trophic polymorphisms [3]. Also, in some freshwater fish populations, trophic polymorphisms are found in relation to different habitat types, such as planktivorous morphs in pelagic habitats and benthivorous morphs in littoral habitats [3, 14, 15]. Discrete habitat specialists usually have divergent traits in trade-offs (when they are tested), e.g., in body shape and head/mouth-part characteristics, that are related to swimming and/or feeding function [5, 6, 16]. If environmental conditions driving natural selection are distinctive and stable, such ecologically important traits are expected to be fixed on an adaptive peak in each habitat [13] and thus lead to phenotypic divergence between environments with assortative mating and reproductive isolation [3, 17]. However, in the wild, the patterns of phenotypic variation and its maintenance mechanisms may change depending on ecological and genetic contexts. Although the underlying mechanisms are poorly documented, traits in divergent selection sometimes present non-discrete (unimodal) variability, including intermediate phenotypes [18, 19].

**Fig. 1:**
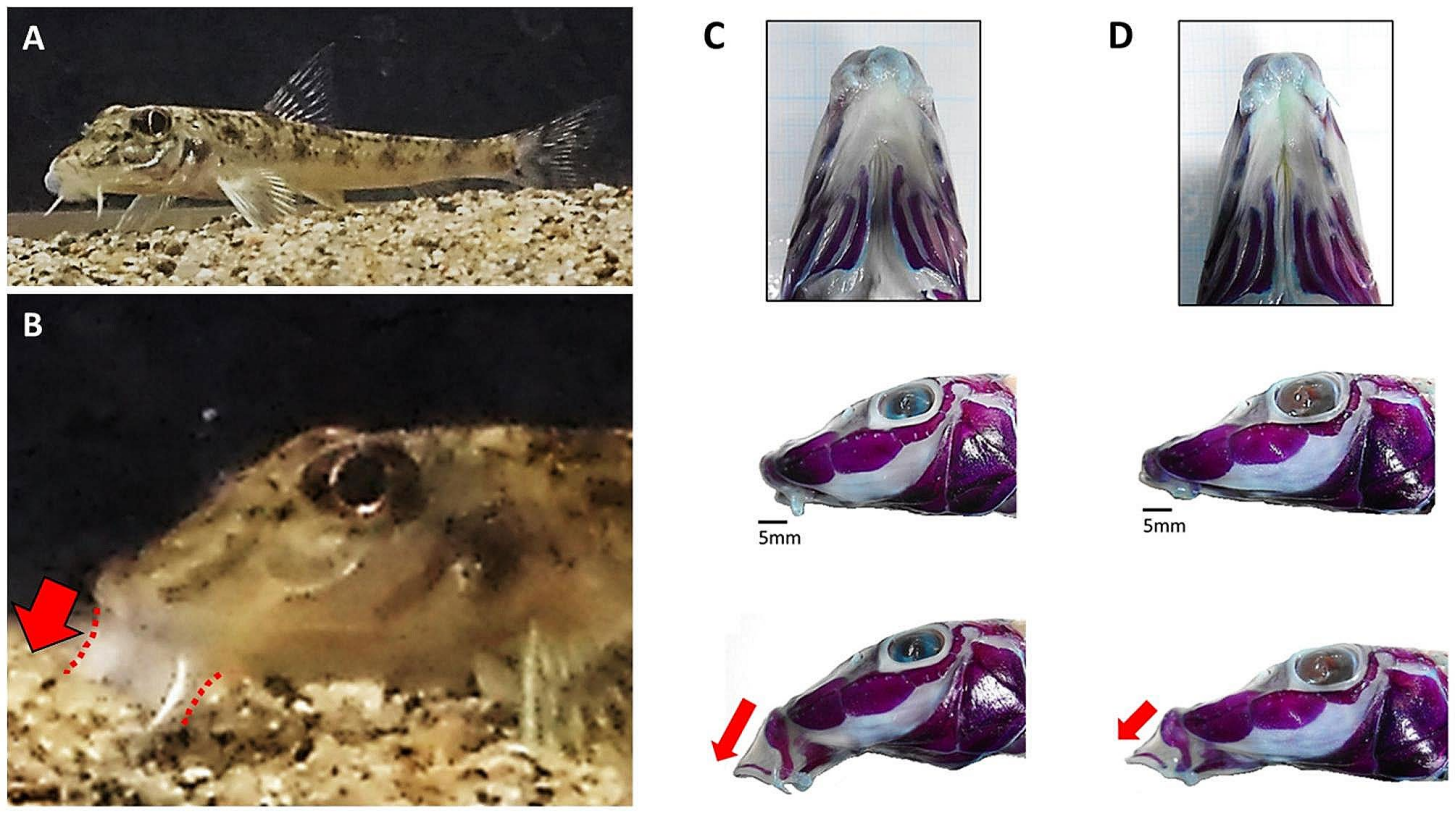
*Pseudogobio esocinus*. (**A**) Resting state, (**B**) feeding state, (**C**) wider mouth, and (**D**) narrower mouth. In the panels C and D, the bones in the head and mouth parts of the fish specimens are stained by Alizarine Red S to visualize anatomical differences. This fish usually exhibits remarkable upper jaw protrusion by opening its mouth to suck prey from the bottom sand (i.e., bottom sucking). It repeats this process by opening and closing its mouth. A wider mouth is linked with markedly downward protrusion, whereas a narrower mouth is linked with more forward and moderate protrusion. Photos (**C**) and (**D**) are reprinted from Endo and Watanabe ([20]; partial modification)

A benthic cyprinid fish, *Pseudogobio esocinus*, in Lake Biwa, central Japan, exhibits a large continuous variation in mouthpart morphology (from wide to narrow) within a lake population [20]. The extreme mouth types are suggested to be important feeding adaptations for their two major prey resources [20]. The fish living in the lake usually eat chironomid larvae, buried prey in the bottom sand, as primary prey. However, compared with the wider-mouthed fish, the narrower-mouthed fish utilize a higher proportion of amphipods, moving prey in the water column, as secondary prey [20]. The mouth shape variation is also linked with kinematic modification of mouth movement (Fig. 1), which presumably contributes to adaptive functional changes to forage/capture the different types of prey [20]. There may be a functional trade-off between mouth types in a large continuous variation, in which individual variation may persist under divergent natural selection for prey usage. However, unlike discrete eco-morphs, the phenotypic variation in the lake population involves neither environmental nor genetic isolation [20]. Thus, how and why such a large non-discrete variability is maintained could not be well explained by the previously known mechanisms. As a first step toward understanding the persistence mechanisms of variability, examining the presence of a functional trade-off in relation to selection pressures within a population should be quite important.

Interestingly, the patterns (extents and mean peaks) of phenotypic variation in the lake population of *P. esocinus* change among sites with different prey/diet contexts [20]. This may imply that different selection pressures act to change their phenotypic distributions depending on local prey conditions (availability). The two major prey resources (i.e., chironomid larvae and amphipods) co-occur even at a single site [21–23], where a correlation between morphology and diet is found [20]. Therefore, the large variability within local populations is a good system to experimentally test a functional trade-off in continuous phenotypic variation using important prey resources as the ecological agents of selection.

Here I aimed to reveal morphology-dependent feeding selectivity and performance with a focus on the presence of a disadvantage in the intermediate phenotype. This was achieved through a laboratory experiment using a variety of individuals caught from a lake site. This paper presents experimental evidence of a potential functional trade-off between phenotypes and feeding efficiency. I discuss the mechanisms of the trade-off in behavioral and functional contexts and the selection pressures that change relative fitness among individuals depending on prey availability.

## Results

**Fig. 2:**
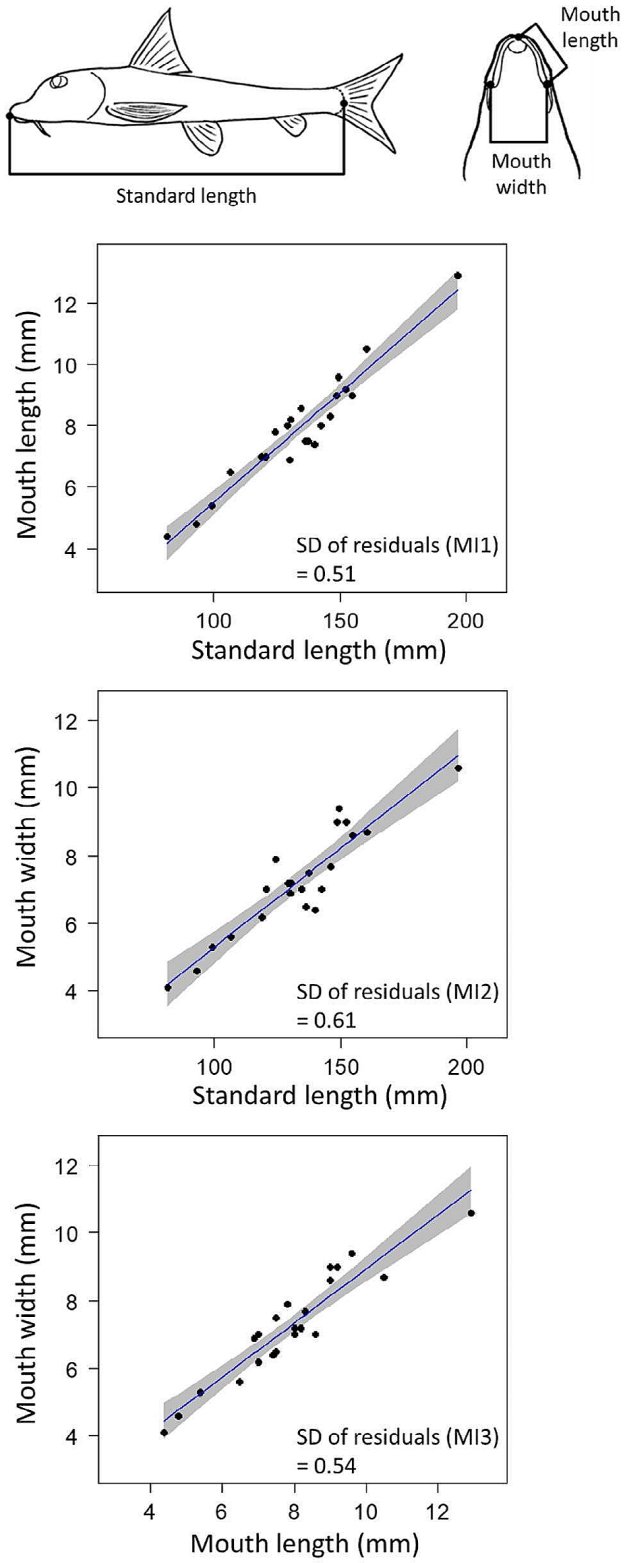
The morphological traits of *Pseudogobio esocinus* focused on in this study and their relationships. Standard length (SL), mouth length (ML), and mouth width (MW). Estimated regression lines between traits (blue lines) with the 95% confidence intervals (gray shades) are shown in the panels. Residuals in the regressions for ML by SL, MW by SL, and MW by ML represent morphological indices (MI1, MI2, and MI3, respectively). Standard deviations (SDs) of MIs are shown on the panels

Prior to the feeding experiment, twenty-two adult fish, whose standard length (SL) was between 81.5 and 196.7 mm, were captured at a single site in Lake Biwa (Wani, Shiga Prefecture, 35.16 N, 135.93E). The fish from this site exhibit a particularly large extent of phenotypic variation in mouthpart morphology [20] (Fig. S1). The twenty-two individuals used for the experiment covered the large mouthpart variation to some degree that was characterized by mouth length (ML) and mouth width (MW) (Fig. 2).

A feeding experiment was conducted to clarify the changes in feeding behavior and feeding efficiency with morphological variation. Each experimental fish was provided the same feeding period (45 min) and same prey availability with two types of prey—100 chironomid larvae buried in the bottom sand (12–15 mm in size and 1.10 mg in average dry weight) and 100 amphipods moving in the water column (5–7 mm in size and 0.83 mg in average dry weight)—a sufficient amount of prey in the feeding period (preliminary observation). Attacking behavior was quantified by counting the number of trials of mouth protrusions (see Fig. 1) during the 45-minute experiment. Selectivity on prey was quantified by counting the number of each type of prey eaten by the fish during the experiment. As feeding efficiency, total food intake in the dry weight (TFI) and efficiency of attacks (total food intake per attack; EA) were also quantified based on these count data (see Materials & Methods).

Since feeding performance may be influenced by the absolute size of the body (SL) and feeding apparatus, I first quantified the overall size of the mouth parts (mouth size) by multiplying the measurements of ML and MW. The measurements of each mouth part and the calculated mouth size were strongly correlated with those of SL due to allometric growth with body size (Pearson’s product-moment correlation tests, ML, *r* = 0.96; MW, *r* = 0.92; mouth size, *r* = 0.94). To test the effects of mouthpart variation on feeding performance independent of those of body size, morphological indices (MIs) for mouthpart variation were defined as residuals from the regression lines for ML to SL (MI1), MW to SL (MI2), and MW to ML (MI3) (Fig. 2).

**Fig. 3:**
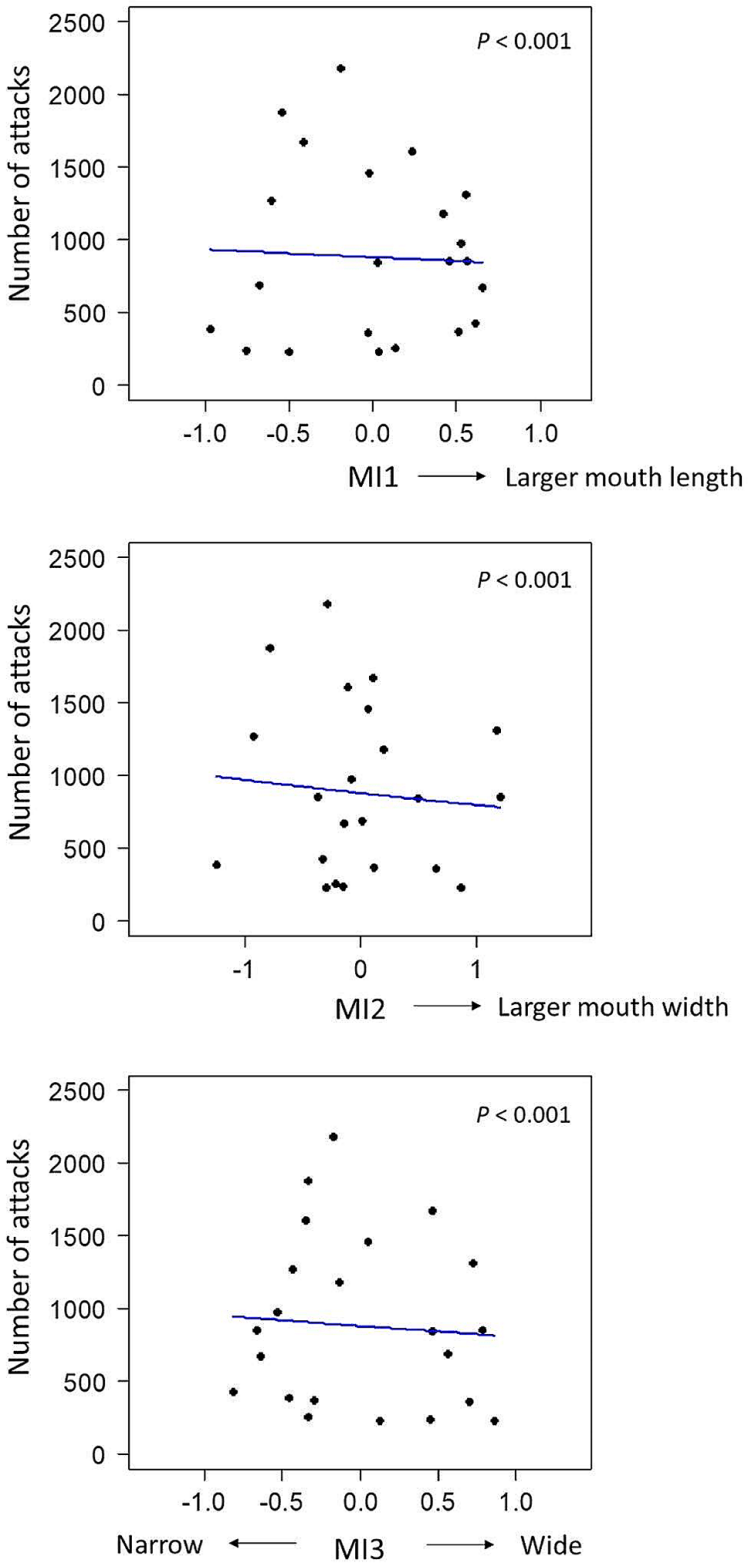
Relationships between morphological characteristics (MIs) and number of attacks for 45 min. Blue lines indicate the regression lines predicted in the GLM multiple regression analysis with standard length as a covariate. MIs represent the morphological indices determined by the residuals in linear regressions for mouth length by standard length (MI1), mouth width by standard length (MI2), and mouth width by mouth length (MI3, i.e., mouth narrowness), respectively

**Fig. 4:**
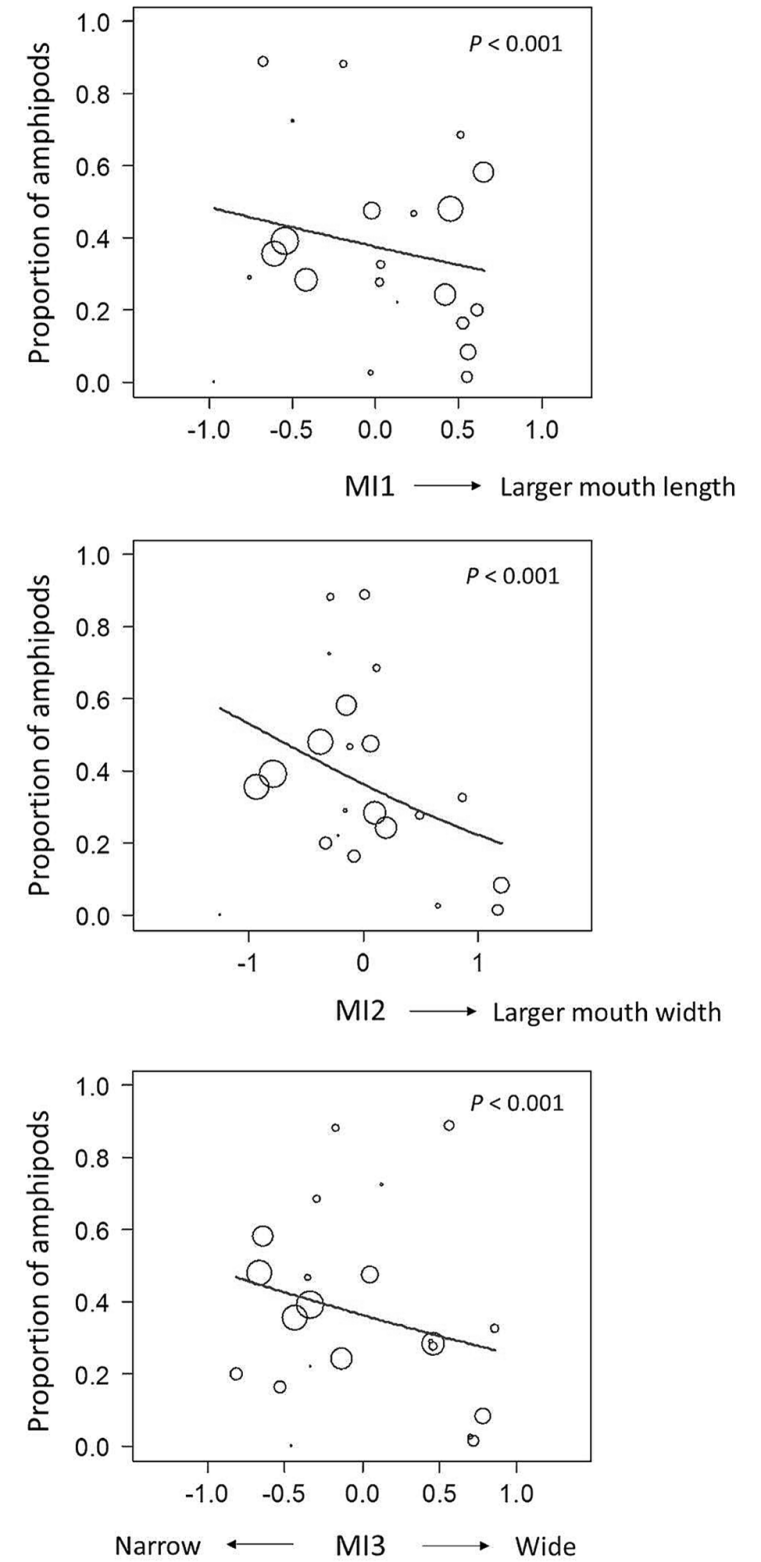
Relationships between morphological characteristics (MIs) and proportion of amphipods. Circle size indicates the relative amount of total prey number the fish ate. Blue lines indicate the regression lines predicted in the GLM multiple regression analysis with standard length as a covariate. MIs represent the morphological indices determined by the residuals in linear regressions for mouth length by standard length (MI1), mouth width by standard length (MI2), and mouth width by mouth length (MI3, i.e., mouth narrowness), respectively

The effects of morphological variation on attacking behavior and prey selectivity were clarified using generalized linear models (GLMs). For attacking behavior, in the simple regression models with either SL or mouth size as an explanatory variable, significant negative effects on the frequency of attacks were found (SL, z = -34.5, *P* < 0.001; mouth size, z = -29.2, *P* < 0.001; Table S1, Fig. S2). In the multiple regression models with each MI index and SL as explanatory variables, the fish with a smaller and narrower mouth exhibited a significantly higher frequency of attacks (MI1, z = -4.02, *P* < 0.001; MI2, z = -7.86, *P* < 0.001; MI3, z = -6.03, *P* < 0.001; Table 1; Fig. 3). For prey selectivity (as proportion of amphipods), in the simple regression models with the same explanatory variables as in tests for attacking behavior, no significant effects of SL or mouth size were found (SL, z = 0.24, *P* = 0.81; mouth size, z = -0.67, *P* = 0.51; Table S1, Fig. S3). In the multiple regression models, the fish with a smaller and narrower mouth exhibited a significantly higher proportion of amphipods (MI1, z = -4.32, *P* < 0.001; MI2, z = -7.42, *P* < 0.001; MI3, z = -5.27, *P* < 0.001; Table 1; Fig. 4).

**Table 1:** Results of GLM multiple regression analyses for frequency of attacks and proportion of amphipods

	Frequency of attacks				Proportion of amphipods		
	Coef.	z	*P*		Coef.	z	*P*
SL	-0.01	-34.5	**< 0.001**		0.001	0.77	0.44
MI1	-0.06	-4.02	**< 0.001**		-0.45	-4.31	**< 0.001**
SL	-0.01	-34.3	**< 0.001**		-0.001	-0.55	0.59
MI2	-0.10	-7.86	**< 0.001**		-0.69	-7.42	**< 0.001**
SL	-0.01	-33.4	**< 0.001**		-0.001	-0.59	0.55
MI3	-0.09	-6.03	**< 0.001**		-0.53	-5.27	**< 0.001**

In the GLMs, standard length (SL) and either one of the morphological indices (MI1–3) were incorporated as explanatory variables. Coefficients (Coef.) indicate the regression estimate values. The z values in tests for frequency of attacks and proportion of amphipods are the statistics in Poisson and binomial models, respectively

**Fig. 5:**
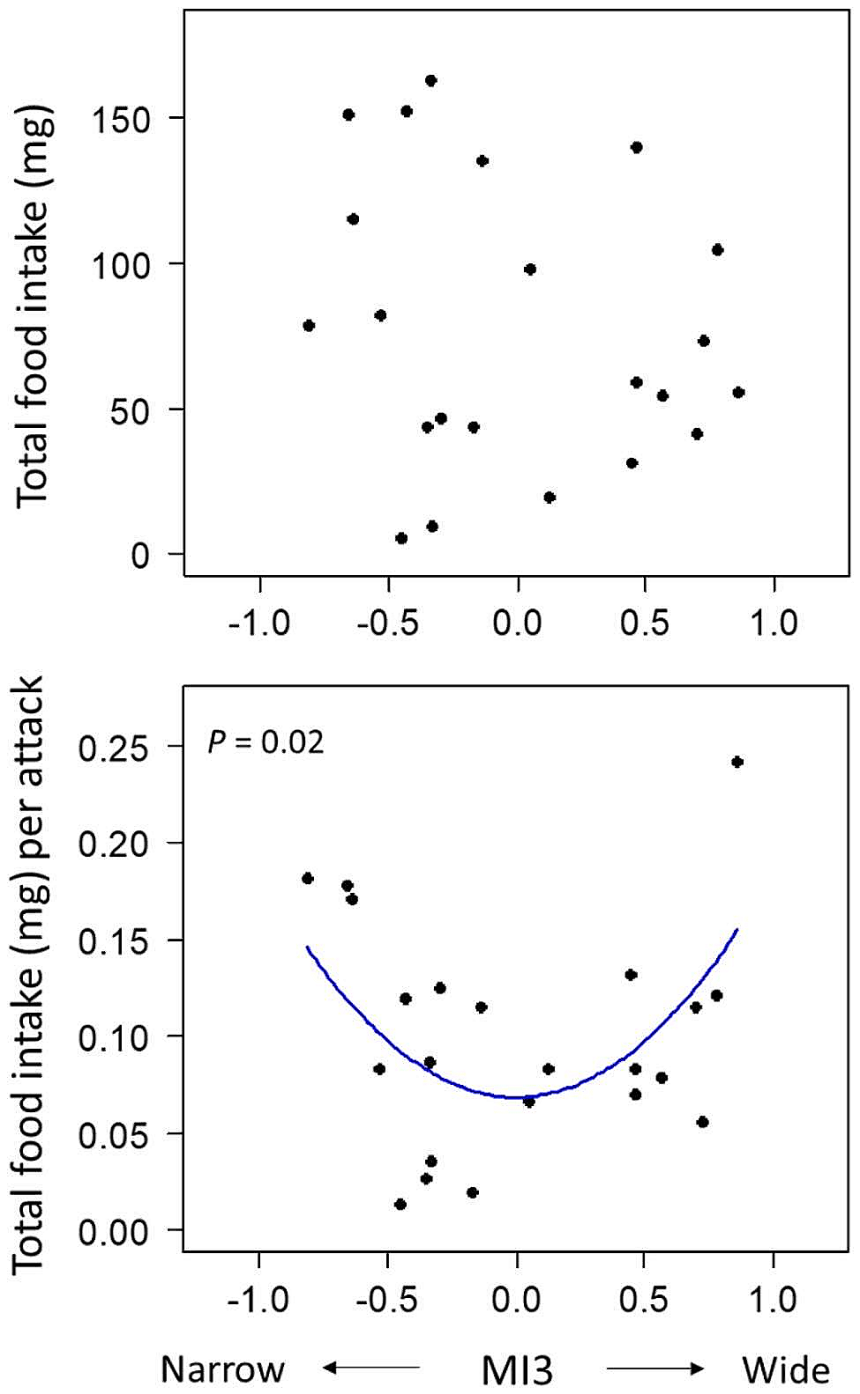
Relationships between MI3 and feeding efficiency as TFI (top) and EA (bottom). The blue line indicates the regression line predicted in the GLM quadratic model (the best model for EA, coefficient of MI3^2^ = 0.12, *P* = 0.02, AIC = -73.16, ΔAIC = 6.48), providing experimental evidence on the potential disadvantage of the intermediate phenotype. MI3 represents the morphological index for mouth narrowness, determined by the residual in the linear regression for mouth width by mouth length

The mouthpart variation also affected the feeding efficiency (as TFI and EA). The absolute body and mouth size affected the total food intake per attack (EA; GLM simple regression, *P* ≤ 0.002) but not the total food intake (TFI; *P* > 0.06; see Table S1, Fig. S4). To detect the most likely relationship between mouthpart variation and feeding efficiency, the two GLMs (for TFI and EA) using each MI index with SL as explanatory variables—the fits of a linear distribution (Efficiency ∼ a + b * SL + c * MI) vs. a quadratic distribution (Efficiency ∼ a’ + b’ * SL + c’ * MI^2^)—were compared based on Akaike information criterion (AIC). This model selection examined the existence of a functional disadvantage of the intermediate mouth type (i.e., a concave quadratic distribution of feeding efficiency along MI) rather than some alternative assumptions (i.e., a convex quadratic- or otherwise a linear distribution). For TFI, neither model was significantly supported in any MI (*P* > 0.3 for MI1, MI1^2^, MI2, MI2^2^, MI3, and MI3^2^; Table S2, Fig. 5). For EA, neither model was significantly supported in MI1 and MI2 (*P* > 0.05 for MI1, MI1^2^, MI2, and MI2^2^; Table 2), but in MI3 (mouth narrowness), the concave quadratic model was significantly supported (coefficient of MI3^2^ = 0.12, t = 2.55, *P* = 0.02) and was the most supportive model (AIC = -73.16, ΔAIC = 6.48; Table 2; Fig. 5).

**Table 2:** Results of model selection in the fits of GLMs for feeding efficiency (as EA)

[Model]:		Coef.	t	*P*	AIC	ΔAIC
[1–1]: Efficiency (EA) ~ SL + MI1	SL	0.001	3.76	**0.001**	-71.08	-2.79
	MI1	0.04	2.05	0.054		
[1–2]: Efficiency (EA) ~ SL + MI1^2^	SL	0.002	3.72	**0.001**	-68.29	
	MI1^2^	-0.05	-1.2	0.24		
[2−1]: Efficiency (EA) ~ SL + MI2	SL	0.001	3.56	**0.002**	-68.69	-0.81
	MI2	0.02	1.35	0.19		
[2–2]: Efficiency (EA) ~ SL + MI2^2^	SL	0.002	3.63	**0.002**	-67.88	
	MI2^2^	-0.02	-1.03	0.31		
[3−1]: Efficiency (EA) ~ SL + MI3	SL	0.001	3.39	**0.003**	-66.68	**6.48**
	MI3	0.001	0.05	0.96		
**[3−2]: Efficiency (EA) ~ SL + MI3** ^**2**^	SL	0.001	2.31	**0.03**	-73.16	
	MI3^2^	0.12	2.55	**0.02**		

The best model for each MI was determined based on Akaike information criterion (AIC). ΔAIC was calculated as the difference between the AIC of the linear model minus the AIC of the quadratic model. Coefficients (Coef.) indicate regression estimate values, and the t values are the statistics in the GLM Gaussian models. A ΔAIC larger than 4 indicates more support for a quadratic model, while a ΔAIC less than − 4 indicates more support for a linear model. A ΔAIC between − 4 and 4 suggests equivalent support between them [33]

## Discussion

### Functional trade-off and its mechanisms

The present experiment clarified that *Pseudogobio esocinus* with diverse mouthpart characteristics exhibited morphology-dependent feeding performance in functional/behavioral contexts. When two types of prey (chironomid larvae and amphipods) were simultaneously provided, the experimental fish more or less ate chironomid larvae, yet the narrower-mouthed fish showed a higher selectivity for amphipods than the wider-mouthed fish (Fig. 4). This is consistent with a field observation of the correlation between morphology and diet at a site in Lake Biwa [20] and suggests that individual morphology contributes to variation in prey selectivity even in a single habitat.

As expected, I found that shape of mouth parts, as well as absolute body and mouth size, were involved in effective feeding for each prey type. Importantly, in the experimental condition with a sufficient amount of both prey items, the intermediate-mouthed fish had lower feeding efficiency (as efficiency of attacks, Fig. 5) than the extreme-mouthed fish. This experimental result supports the presence of a functional trade-off and potential disadvantage of the intermediate phenotype. Feeding efficiency as total food intake itself was not found to be changed along with morphological variation in this experiment. This could possibly be due to that the effort for attacking trials was affected by some capricious factors of individuals, and especially, the intermediate phenotype might try to attack more frequently to compensate for the lower efficiency of attacks for both prey types compared with that of extreme phenotypes. Even if so, the intermediate phenotype could have adverse fitness consequences due to the higher energy costs in increasing attacking trials.

The experimental fish took prey by bottom sucking as its basic feeding (attacking) behavior, which decreased in frequency as body and mouth size increased (Fig. S2). Not only that, the wider-mouthed fish, which consumed more chironomid larvae (Fig. 4), showed less frequent attacks than the narrower-mouthed fish (Fig. 3). However, the feeding efficiency of the former (TFI, EA) did not decrease compared to that of the latter (Fig. 5). It is inferred that the wider-mouthed fish may perform more effective bottom sucking at the sacrifice of frequency of the activity and the inferred improvement in the efficiency of attacks may be attributed to a higher consumption rate of chironomid larvae. To efficiently suck and collect buried prey, such as chironomid larvae, a stronger suction force for digging up the bottom sand is important [24, 25]. For this reason, the wider-mouthed fish have advantages because a wider mouth generates an increased suction force by the larger buccal cavity than a narrower mouth [24–26]. Additionally, a wider mouth is linked with more downward mouth protrusion (Fig. 1; [20]), which can enhance the functional advantage of the wider-mouthed fish in increasing suction force and reaching out further for buried prey.

On the other hand, the narrower-mouthed fish, which consumed more amphipods (Fig. 4), exhibited more frequent attacks than the wider-mouthed fish (Fig. 3). To capture moving/escaping prey such as amphipods more efficiently, the fish may have to attack them more quickly. In addition, compared with the wider-mouthed fish, the narrower-mouthed fish would be forced to decrease the suction force of an attack due to the smaller buccal cavity and shorter/moderate mouth protrusion (Fig. 1; [20]). Instead of such behavioral/functional demands and costs, however, the narrower-mouthed fish improved their feeding efficiency to approximately equal to that of the wider-mouthed fish (Fig. 5). Therefore, they are likely to have some advantages as improving feeding efficiency in an alternative way for better use of their unique mouth parts (Fig. 1). For example, when the narrower-mouthed fish aim at amphipods just in front of the mouth, the narrower/smaller mouth parts with more forward and moderate mouth protrusion may be convenient for performing a quick and short attack [20, 24, 25, 27]. As a result, the modified feeding behavior by the narrower-mouthed fish may work to increase the capture rate on amphipods by improving the efficiency of attacks.

An important limitation of this experiment was that it was not possible to compare attack success rates for each prey type because both prey items (chironomid larvae and amphipods) were provided simultaneously and the target for each attack could not be specified. This made it difficult to strictly distinguish whether the resulting feeding efficiency associated with the mouthpart morphology was due to differences in prey selectivity or to differences in attack success rates unrelated to the selectivity. To verify the effects of functional trade-offs separated from those of prey selectivity, an additional experiment quantifying feeding performance for each prey type is necessary.

### Impacts of a trade-off on persistence mechanisms of variability

Through consistent feeding adaptations in morphology and behavior, both extreme mouth types could improve relative fitness as long as their preferrable prey resources are sufficiently available, as in this experiment. Although the nutritional values of those prey items for this fish are unknown, if a certain prey context provides an equivalent benefit for both mouth types, they could coexist even in a habitat [28]. On the other hand, when only one type of prey, i.e., either buried or moving prey, is available, the fish with more adaptive/specialized mouth parts for the prey type, i.e., extremely a wider- or a narrower-mouth type, would be favored in the habitat. Furthermore, the fitness costs of phenotypes may also be changed according to the relative density of mouth types that explore the same prey resources in their habitat [11, 28]. In that way, the patterns of phenotypic distributions can vary depending on local prey contexts under disruptive, directional, or frequency-dependent selection.

Now, in the presence of a functional trade-off and potential disadvantage of an intermediate phenotype, why does the least efficient phenotype persist? The current experiment only clarified the disadvantage of the intermediate phenotype when both prey items were available. However, even if either type of prey is available, the intermediate phenotype may also suffer higher fitness costs compared with the extreme (more adaptive) phenotype due to the potential fitness costs of the trade-off. Therefore, the intermediate phenotype could have difficulty persisting/coexisting with extreme phenotypes, although other factors may also influence the outcome of the relative fitness [28]. Unfortunately, the genetic background (or phenotypic plasticity) underlying mouthpart variability is unknown, and thus, sufficient knowledge is lacking to determine the evolutionary impacts of the trade-off on the maintenance mechanisms of variability. However, one possible explanation may be that the intermediate phenotype is inevitably produced with ongoing selection under genetic/reproductive constraints to prevent phenotypic divergence. If ecologically driven trait variability is not accompanied by assortative mating, recombination in the trait under divergent selection may produce unimodal/non-discrete phenotypic variation [19, 29]. Additionally, there may be some ecological situations of relaxing trade-offs; that is, generalizations rather than specializations can often be predicted to be favored when individuals are forced to perform a range of activities under temporally fluctuating environments [28]. In fact, in the wild, the abundance of amphipods at the lake sites varies markedly with the season ( [22]; personal observation). In such situations, both extreme and intermediate phenotypes may coexist [28, 30], and high individual variability should contribute to this fish population persisting with flexible responses to a wide variety of environmental (prey) conditions.

## Conclusions

I experimentally clarified the existence of feeding selectivity and a functional trade-off associated with continuous phenotypic variation, including an intermediate phenotype, in a single population. This result suggests that diverse individuals exhibit morphology-dependent feeding performance even under the same environmental (prey) conditions. In this context, the acts of divergent selection depending on prey availability and impacts of the trade-off on some possible mechanisms of the coexistence of both extreme and intermediate phenotypes were discussed. Although further examinations, especially focusing on feeding efficiency for each prey type separated from the effects of prey selectivity, are needed, this case represents an opportunity to consider the mechanisms of the persistence of phenotypic variation that is maintained without divergence even in a trade-off.

## Materials and methods

### Fish sampling

For the feeding experiment, fish sampling was conducted at a single site in Lake Biwa (Wani, Shiga Prefecture, 35.16 N, 135.93E), where a particularly large extent of mouthpart variation in *Pseudogobio esocinus* was found (Fig. S1; [20]). The sampling was carried out using a cast net (for adult fish size) between July and August in 2016. All actively living individuals—twenty-two adult fish (i.e., one year old or older based on body size [31]) with a standard length between 81.5 and 196.7 mm—were used in the experiment after being kept on an empty stomach for 1 day before the experiment.

### Feeding experiment

I conducted the experiment for individual fish under the same feeding period (for 45 min) and same prey condition. A fish was simultaneously provided two types of prey—100 chironomid larvae and 100 amphipods—a sufficient amount of prey in the feeding period (preliminary observation). The experimental tank was 400 mm long, 200 mm wide, and 300 mm in water depth with a 30 mm layer of bottom sand. The grain size of the bottom sand was made finer than 2 mm through a sieve. For diets of *P. esocinus* in Lake Biwa, chironomid larvae are categorized as a type of buried prey in the bottom sand (low mobility), whereas amphipods are a type of moving prey in the water column [20]. In the habitats of the fish in Lake Biwa (sandy and pebbly zones), many large chironomid larvae, including *Chironomus*, are buried in the bottom sand. Additionally, four amphipod species, including *Crangonyx floridanus* (an alien species), inhabit and actively move on the bottom substrates, in the vegetation area, and throughout the water column ( [21, 22]; personal observation). I tried to replicate the wild prey conditions, especially in prey types and size classes (based on personal observation), as much as possible. Chironomid larvae (commercially obtained from KYORIN CO., LTD, dead individuals as larvae of *Chironomus* that were 12–15 mm in size and 1.10 mg in average dry weight) were buried in the bottom sand, where the fish ate even dead chironomids. Amphipods (wild-caught from Lake Biwa, living individuals of *Crangonyx floridanus* that were 5–7 mm in size and 0.83 mg in average dry weight) were placed in the water column, where they moved actively.

Once a fish was placed in the experimental tank, it remained motionless for a while. After the fish started foraging (if early, in a few minutes, at latest, in a half-hour), I observed and recorded its feeding activity for 45 min. I visually counted the number of trials of attacking behavior (mouth protrusions, see Fig. 1) during the experimental period and also checked it with the video data. After the experiment, I counted the number of each type of prey that remained in the tank to calculate the total prey number eaten by the fish. The feeding efficiency of the fish was defined as total food intake (TFI; sum of the weight of each type of prey that was calculated by multiplying the average prey weight and the number) and efficiency of attacks (EA; total food intake per attack).

### Morphological measurements

Following the experiment, the fish were placed in a bucket with a small amount of water, just enough to cover the fish’s body, to which a few drops of an anesthetic (2-phenoxyethanol) were added (at a concentration sufficient to sedate an adult fish within minutes). After the fish became comatose, they were promptly transferred onto ice for euthanasia. According to Endo & Watanabe [20], I measured the three important morphological traits (distances between selected points) on the fish body by vernier caliper to the nearest 0.1 mm: standard length (SL), mouth length (ML), and mouth width (MW) (Fig. 2). Since feeding performance may be influenced by the absolute size of the body and feeding apparatus, I first quantified the overall size of the mouth parts by multiplying the measurements of ML and MW. Since allometric growth were expected, I first examined the correlation between mouth size and SL. To obtain morphological indices (MIs) for mouthpart variation independent of body size, I calculated the residuals in the linear regressions for ML by SL (MI1), MW by SL (MI2), and MW by ML (the proportion of mouth parts, i.e., narrowness; MI3) (Fig. 2).

### Statistical analyses

To test the effects of morphological variation on attacking behavior, I conducted generalized linear models (GLMs) for the frequency of attacks (the number of attacks for 45 min) with a Poisson distribution. As a preliminary analysis, simple regression models with SL or mouth size as an explanatory variable were examined. Then, considering the effects of body size (that related to mouth size), the multiple regression models, which were incorporated with SL and either of the MI indices as explanatory variables, were analyzed. Similarly, to test the effects of morphological variation on prey selectivity, the GLM simple and multiple regression analyses for proportion of amphipods (in the total prey number) were conducted with a binomial distribution using the same explanatory variables as in tests for the frequency of attacks.

To examine the effects of morphological variation on feeding efficiency (as TFI and EA, respectively), the GLM regression analysis was conducted with a Gaussian distribution using the same explanatory variables as the above tests. Particularly, to examine whether the intermediate phenotype exhibits a disadvantage in feeding efficiency, I determined the most likely relationship between MIs and feeding efficiency (as TFI and EA, respectively) through the following model selection. I compared the fits of two GLMs with a linear distribution (as an alternative hypothesis) versus a quadratic distribution (as a model for the disadvantage of the intermediate phenotype). For the model selection, the values of Akaike information criterion (AIC) and ΔAIC as the difference between the AIC of the linear model minus the AIC of the quadratic model were calculated [32]. A ΔAIC larger than 4 was interpreted as indicating more support for a quadratic model, while a ΔAIC less than − 4 indicated more support for a linear model. A ΔAIC between − 4 and 4 was interpreted as showing equivalent support between them [33]. All correlation tests in the GLMs were performed by using the glm function in R software ver. 3.5.0 (R Core Team 2018).

### Electronic supplementary material

Below is the link to the electronic supplementary material.

Supplementary Material 1

## Data Availability

All data analyzed during this study are included in this published article and its supplementary information files or are available from the corresponding author upon reasonable request.

## Abbreviations

SL: Standard length
ML: Mouth length
MW: Mouth width
TFI: Total food intake
EA: Efficiency of attacks
MIs: Morphological indices
GLMs: Generalized linear models
AIC: Akaike information criterion
Coef.: Coefficient
SDs: Standard deviations

## References

[1] Van Valen L (1965). Morphological variation and width of ecological niche. Am Nat.

[2] Grant PR, Price TD (1981). Population variation in continuously varying traits as an ecological genetics problem. Am Zool.

[3] Smith TB, Skúlason S (1996). Significance of resource polymorphisms in fishes, amphibians, and birds. Annu Rev Ecol Syst.

[4] Bolnick DI, Svanbäck R, Fordyce JA, Yang LH, Davis JM, Hulsey CD (2003). The ecology of individuals: incidence and implications of individual specialization. Am Nat.

[5] Schluter D (1993). Adaptive radiation in sticklebacks: size, shape, and habitat use efficiency. Ecology.

[6] Schluter D (1995). Adaptive radiation in stickleback: trade-offs in feeding performance and growth. Ecology.

[7] Raimchen TE, Nosil P (2002). Temporal variation in divergent selection on spine number in a population of threespine stickleback. Evolution.

[8] Grant PR, Grant BR (2002). Unpredictable evolution in a 30-year study of Darwin’s finches. Science.

[9] Dewitt TJ, Langerhans RB. Integrated solutions to environmental heterogeneity. In: Dewitt TJ, Scheiner SM, editors. Phenotypic plasticity: functional and conceptual approaches. Oxford University Press; 2004. pp. 98–111.

[10] Johnson JB, Burt DB, Dewitt TJ (2008). Form, function, and fitness: pathways to survival. Evolution.

[11] Futuyma DJ, Moreno G (1988). The evolution of specialization. Annu Rev Ecol Syst.

[12] Arnold SJ (1983). Morphology, performance and fitness. Integr Comp Biol.

[13] Endler JA. Natural selection in the wild. Princeton University Press; 1986.

[14] Robinson BW, Wilson DS (1994). Character release and displacement in fishes: a neglected literature. Am Nat.

[15] Skúlason S, Smith TB (1995). Resource polymorphisms in vertebrates. Trends Ecol Evol.

[16] Svanbäck R, Eklöv P (2003). Morphology dependent foraging efficiency in perch: a trade-off for ecological specialization?. Oikos.

[17] Siepielski AM, Gotanda KM, Morrissey MB, Diamond SE, DiBattista JD, Carlson SM (2013). The spatial patterns of directional phenotypic selection. Ecol Lett.

[18] Robinson BW, Wilson DS, Shea GO (1996). Trade-offs of ecological specialization: an intraspecific comparison of pumpkinseed sunfish phenotypes. Ecology.

[19] Snowberg LK, Bolnick DI (2008). Assortative mating by diet in a phenotypically unimodal but ecological variable population of stickleback. Am Nat.

[20] Endo C, Watanabe K (2020). Morphological variation associated with trophic niche expansion within a lake population of a benthic fish. PLoS ONE.

[21] Nishino M (1992). Benthos of Lake Biwa–riparian lives– aquatic insect’s series.

[22] Nishino M (1993). Benthos of Lake Biwa–riparian lives– crustacean’s series.

[23] Nishino M (2000). The community of benthos in Lake Biwa. Kaiyo Monthly.

[24] Wassenbergh SV, Aerts P, Harrel A (2006). Scaling of suction feeding performance in the catfish *Clarias gariepinus*. Physiol Biochem Zool.

[25] Wainwright PC, Carroll AM, Collar DC, Day SW, Higham TE, Holzman RA (2007). Suction feeding mechanisms, performance, and diversity in fishes. Integr Comp Biol.

[26] Alexander RMcN (1966). The functions and mechanisms of the protrusible upper jaws of two species of cyprinid fish. J Zool Lond.

[27] Holzman R, Collar DC, Mehta RS, Wainwright PC (2011). Functional complexity can mitigate performance trade-offs. Am Nat.

[28] Wilson DS, Yoshimura J (1994). On the coexistence of specialists and generalists. Am Nat.

[29] Bolnick DI (2006). Multi-species outcomes of a common model of sympatric speciation. J Theor Biol.

[30] Egas M, Dieckman U, Sabelis MW (2004). Evolution restricts the coexistence of specialists and generalists: the role of trade-off structure. Am Nat.

[31] Nakamura M (1969). Cyprinid fishes of Japan.

[32] Burnham KP, Anderson DR (2002). Model selection and inference: a practical information-theoretic approach.

[33] Burnham KP, Anderson DR, Huyvaert KP (2011). AIC model selection and multimodel inference in behavioral ecology: some background, observations, and comparisons. Behav Ecol Sociobiol.

