# Supplementary material for: Feeding selectivity and a functional trade-off in a benthic fish with a continuous morphological variation: an experimental test
Source: BMC Zool. 2024 Mar 4;9:5. doi: 10.1186/s40850-024-00194-z (PMC10910733; doi:10.1186/s40850-024-00194-z)
Supplement: Supplementary file 1 — Supplementary Material 1 [file 40850_2024_194_MOESM1_ESM.pdf]

Supplementary information for

Feeding selectivity and a functional trade-off in a benthic fish with a continuous morphological variation: an experimental test

Chiharu Endo

Laboratory of Animal Ecology, Graduate School of Science, Kyoto University

Laboratory of Forest Biology, Graduate School of Agriculture, Kyoto University, Kitashirakawa Oiwake-cho, Sakyo-ku, Kyoto, 606-8502, Japan (present address)

Correspondence and request for materials should be addressed to C.E.

Email: chiharupn1622@gmail.com

Contents:

S1–S4 Figures

S1, S2 Tables

References

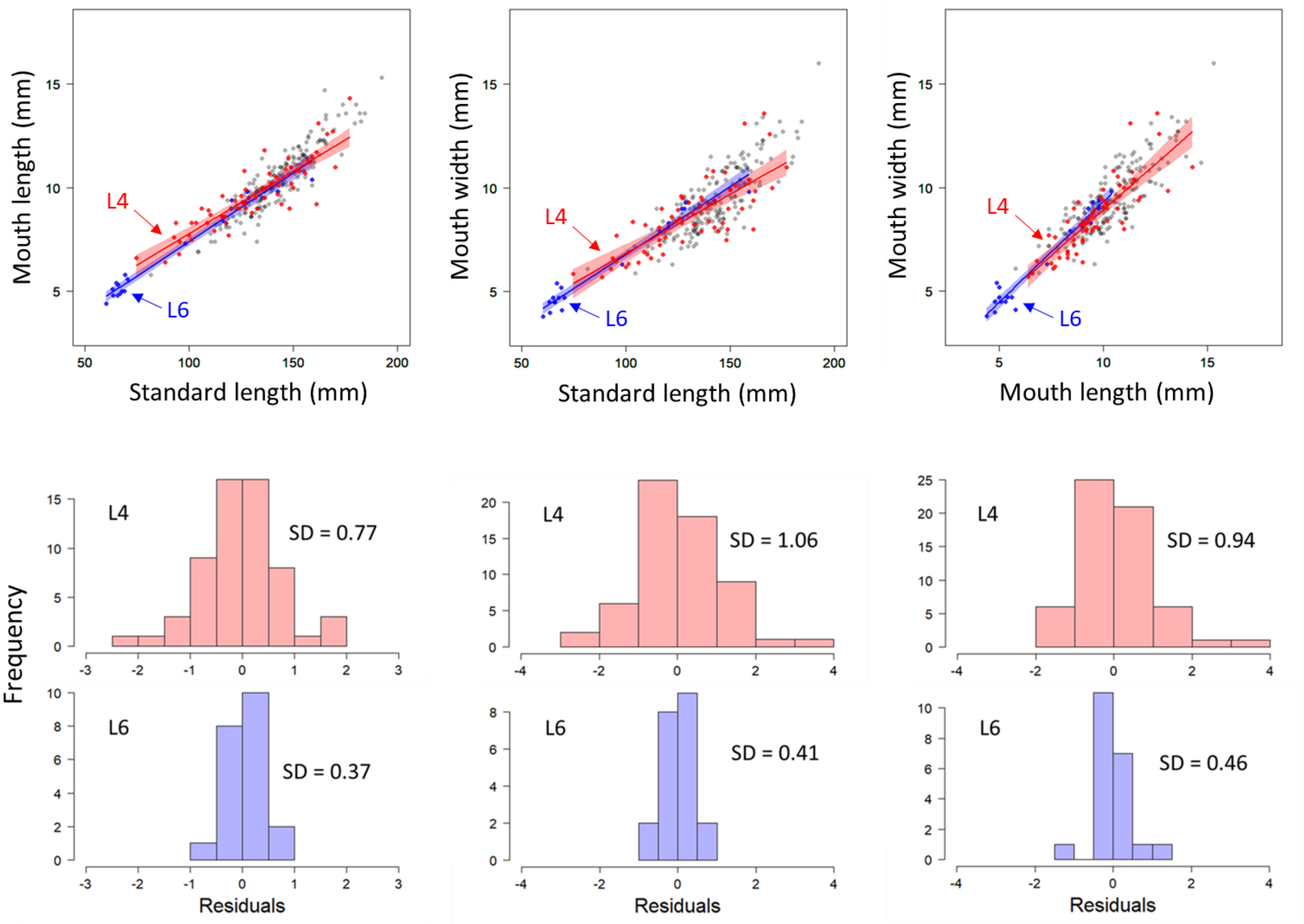
Mouth length (mm)
Standard length (mm)
L4
L6
Mouth width (mm)
Standard length (mm)
L4
L6
Mouth width (mm)
Mouth length (mm)
L4
L6
Frequency
L4
SD = 0.77
L6
SD = 0.37
Residuals
L4
SD = 1.06
L6
SD = 0.41
Residuals
L4
SD = 0.94
L6
SD = 0.46
Residuals

Figure S1. Relationships between morphological traits focused on in this study and histograms of residuals in the linear regression analysis for *Pseudogobio esocinus* from the representative localities in Lake Biwa. Red plots indicate the locality sample in L4 (Wani), where the fish exhibit an extremely high trait variability. Blue plots indicate the sample in L6, where the fish show a relatively low variability. Grey plots are of the other locality samples in Lake Biwa (see [1]). Estimated regression lines between traits (red/blue lines) with the 95% confidence intervals (red/blue shades) are shown in the upper panels. Standard deviations (SDs) of the regressions are shown on the histograms. All the data sets are from Endo and Watanabe [1].

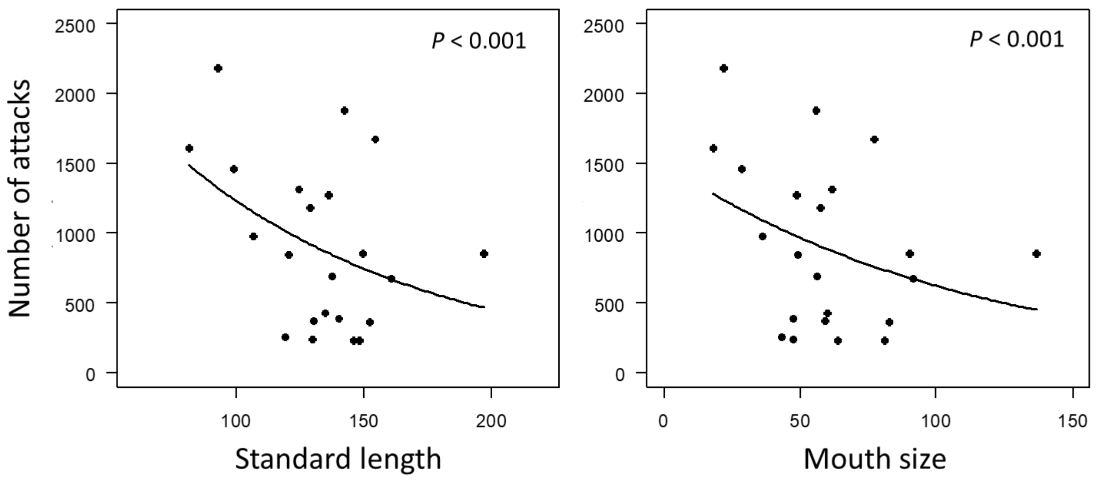

Figure S2. Relationships between morphological characteristics (standard length and mouth size) and number of attacks for 45 minutes. The frequency of attacks significantly decreased with increasing body size and mouth size (GLM, standard length, $z = -34.5$, $P < 0.001$; mouth size, $z = -29.2$, $P < 0.001$).

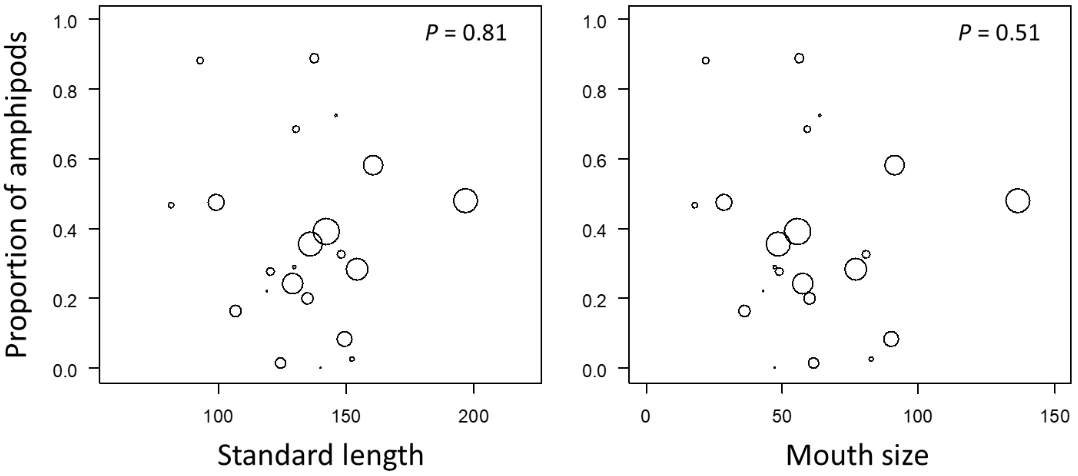

Figure S3. Relationships between morphological characteristics (standard length and mouth size) and proportion of amphipods. Circle size indicates the relative amount of total prey number the fish ate. There was no significant effect of body size nor mouth size on the proportion of amphipods (GLM, standard length, z = 0.24, *P* = 0.81; mouth size, z = -0.67, *P* = 0.51).

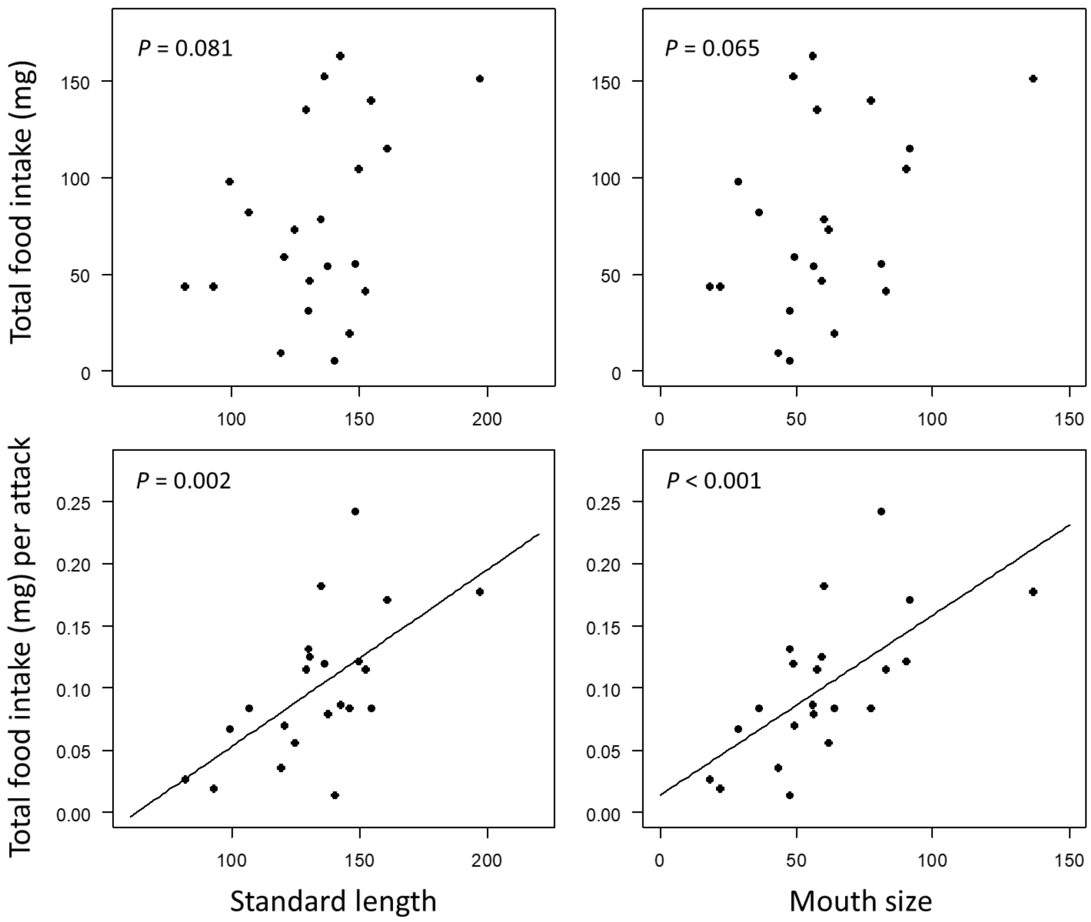

Figure S4. Relationships between morphological characteristics (standard length and mouth size) and feeding efficiency as total food intake, TFI (top) and efficiency of attacks, EA (bottom). The feeding efficiency (as EA) significantly improved with increasing body size and mouth size (GLM, standard length, t = 3.49, $P = 0.002$; mouth size, t = 3.98, $P < 0.001$), but the effects on TFI were not significant (standard length, t = 1.84, $P = 0.081$; mouth size, t = 1.95, $P = 0.065$).

Table S1. Results of GLM simple regression analyses for frequency of attacks, proportion of amphipods, and feeding efficiency (TFI, EA).

	Frequency of attacks			Proportion of amphipods			Feeding efficiency (TFI)			Feeding efficiency (EA)		
	Coef.	z	*P*	Coef.	z	*P*	Coef.	t	*P*	Coef.	t	*P*
Standard length	-0.01	-34.5	< **0.001**	0.0005	0.24	0.81	0.74	1.84	0.081	0.001	3.49	**0.002**
Mouth size	-0.009	-29.2	< **0.001**	-0.001	-0.67	0.51	0.73	1.95	0.065	0.001	3.98	< **0.001**

In the GLMs, standard length or mouth size was incorporated as an explanatory variable. Coefficients (Coef.) indicate the regression estimate values. The z values in tests for frequency of attacks and proportion of amphipods are the statistics in Poisson and binomial models, respectively. The t values in tests for TFI and EA are the statistics in Gaussian models.

Table S2. Results of model selection in the fits of GLMs for feeding efficiency (as TFI).

[Model]:		Coef.	t	*P*	AIC	ΔAIC
[1-1]: Efficiency (TFI) ~ SL + MI1	SL	0.74	1.84	0.08	235.80	-0.14
	MI1	18.7	0.95	0.35		
[1-2]: Efficiency (TFI) ~ SL + $MI1^2$	SL	0.84	2.00	0.06	235.94	
	$MI1^2$	-40.3	-0.88	0.39		
[2-1]: Efficiency (TFI) ~ SL + MI2	SL	0.74	1.80	0.09	235.73	0.02
	MI2	-4.66	-0.28	0.78		
[2-2]: Efficiency (TFI) ~ SL + $MI2^2$	SL	0.77	1.82	0.08	235.71	
	$MI2^2$	-6.35	-0.31	0.76		
[3-1]: Efficiency (TFI) ~ SL + MI3	SL	0.76	1.90	0.07	235.55	-0.92
	MI3	-19.9	-1.06	0.30		
[3-2]: Efficiency (TFI) ~ SL + $MI3^2$	SL	0.86	1.86	0.08	236.47	
	$MI3^2$	-28.6	-0.55	0.59		

The best model for each morphological index (MI) was determined based on Akaike information criterion (AIC). ΔAIC was calculated as the difference between the AIC of the linear model minus the AIC of the quadratic model. Coefficients (Coef.) indicate regression estimate values, and the t values are the statistics in the GLM Gaussian models. A ΔAIC larger than 4 indicates more support for a quadratic model, while a ΔAIC less than -4 indicates more support for a linear model. A ΔAIC between -4 and 4 suggests equivalent support between them [2]. SL stands for standard length, and MIs represent the morphological indices determined by the residuals in linear regressions for mouth length by SL (MI1), mouth width by SL (MI2), and mouth width by mouth length (MI3, i.e., mouth

narrowness), respectively.

References:

1. Endo C, Watanabe K. Morphological variation associated with trophic niche expansion within a lake population of a benthic fish. PLoS ONE 2020;15:e0232114. doi: 10.1371/journal.pone.0232114

2. Burnham KP, Anderson DR, Huyvaert KP. AIC model selection and multimodel inference in behavioral ecology: some background, observations, and comparisons. Behav. Ecol. Sociobiol. 2011;65:23-35
